# Supplementary material for: Modeling glioblastoma heterogeneity as a dynamic network of cell states
Source: Mol Syst Biol. 2021 Sep 16;17(9):e10105. doi: 10.15252/msb.202010105 (PMC8444284; doi:10.15252/msb.202010105)
Supplement: Supplementary file 6 — Source Data for Figure 5 [file MSB-17-e10105-s004.zip › Figure5A_sourcedata/GSEA_3017/nosigdb_stateF.GseaPreranked.1622795015644/RICHARDS_NOWAKOWSKI_SCIENCE_2017_MGE_DIV_UPREG.html]

Details for gene set RICHARDS\_NOWAKOWSKI\_SCIENCE\_2017\_MGE\_DIV\_UPREG[GSEA]

|  || Dataset | state33017 |
| Phenotype | NoPhenotypeAvailable |
| Upregulated in class | na\_neg |
| GeneSet | RICHARDS\_NOWAKOWSKI\_SCIENCE\_2017\_MGE\_DIV\_UPREG |
| Enrichment Score (ES) | -0.7487332 |
| Normalized Enrichment Score (NES) | -5.968079 |
| Nominal p-value | 0.0 |
| FDR q-value | 0.0 |
| FWER p-Value | 0.0 |
Table: GSEA Results Summary

  

Fig 1: Enrichment plot: RICHARDS\_NOWAKOWSKI\_SCIENCE\_2017\_MGE\_DIV\_UPREG      
 Profile of the Running ES Score & Positions of GeneSet Members on the Rank Ordered List

  

| PROBE | GENE SYMBOL | GENE\_TITLE | RANK IN GENE LIST | RANK METRIC SCORE | RUNNING ES | CORE ENRICHMENT || 1 | FBXW7 |  |  | 272 | 0.537 | -0.1392 | No |
| 2 | SEMA5A |  |  | 336 | 0.494 | -0.1697 | No |
| 3 | ABAT |  |  | 362 | 0.483 | -0.1803 | No |
| 4 | HSPA5 |  |  | 373 | 0.480 | -0.1832 | No |
| 5 | ALDOA |  |  | 377 | 0.479 | -0.1825 | No |
| 6 | KITLG |  |  | 401 | 0.470 | -0.1922 | No |
| 7 | TMSB4X |  |  | 590 | 0.401 | -0.2882 | No |
| 8 | GRIK3 |  |  | 598 | 0.399 | -0.2900 | No |
| 9 | NDUFB3 |  |  | 629 | 0.389 | -0.3037 | No |
| 10 | BCHE |  |  | 677 | 0.376 | -0.3264 | No |
| 11 | SH3BGRL |  |  | 695 | 0.373 | -0.3334 | No |
| 12 | PFN2 |  |  | 724 | 0.365 | -0.3463 | No |
| 13 | MSI2 |  |  | 780 | 0.352 | -0.3732 | No |
| 14 | RAB3IP |  |  | 797 | 0.348 | -0.3799 | No |
| 15 | AGL |  |  | 976 | 0.314 | -0.4712 | No |
| 16 | SRGAP2B |  |  | 1006 | 0.309 | -0.4848 | No |
| 17 | ANKRD50 |  |  | 1084 | 0.295 | -0.5235 | No |
| 18 | SPECC1 |  |  | 1089 | 0.294 | -0.5242 | No |
| 19 | NELL2 |  |  | 1159 | 0.284 | -0.5588 | No |
| 20 | CKB |  |  | 1200 | 0.279 | -0.5783 | No |
| 21 | COX6C |  |  | 1252 | 0.274 | -0.6035 | No |
| 22 | GAPDH |  |  | 1361 | 0.261 | -0.6586 | No |
| 23 | DCHS1 |  |  | 1411 | 0.255 | -0.6829 | No |
| 24 | FANCL |  |  | 1442 | 0.253 | -0.6973 | No |
| 25 | PCDH19 |  |  | 1460 | 0.251 | -0.7049 | No |
| 26 | RFC5 |  |  | 1473 | -0.250 | -0.7100 | No |
| 27 | DNA2 |  |  | 1479 | -0.252 | -0.7114 | No |
| 28 | TARDBP |  |  | 1480 | -0.252 | -0.7101 | No |
| 29 | SNRPG |  |  | 1486 | -0.254 | -0.7115 | No |
| 30 | RPA2 |  |  | 1490 | -0.256 | -0.7118 | No |
| 31 | HNRNPA2B1 |  |  | 1503 | -0.261 | -0.7168 | No |
| 32 | BLM |  |  | 1507 | -0.262 | -0.7171 | No |
| 33 | BRD8 |  |  | 1508 | -0.263 | -0.7158 | No |
| 34 | HCFC1 |  |  | 1515 | -0.264 | -0.7177 | No |
| 35 | MSH6 |  |  | 1517 | -0.264 | -0.7169 | No |
| 36 | RBBP8 |  |  | 1518 | -0.265 | -0.7156 | No |
| 37 | ZNF43 |  |  | 1544 | -0.270 | -0.7274 | No |
| 38 | BAZ1A |  |  | 1547 | -0.271 | -0.7271 | No |
| 39 | FAM76B |  |  | 1552 | -0.272 | -0.7278 | No |
| 40 | HNRNPUL2 |  |  | 1554 | -0.273 | -0.7270 | No |
| 41 | PSMB6 |  |  | 1558 | -0.274 | -0.7273 | No |
| 42 | NUP62 |  |  | 1562 | -0.275 | -0.7275 | No |
| 43 | PSIP1 |  |  | 1564 | -0.275 | -0.7267 | No |
| 44 | NAP1L4 |  |  | 1568 | -0.277 | -0.7269 | No |
| 45 | WDHD1 |  |  | 1569 | -0.277 | -0.7256 | No |
| 46 | MRPL51 |  |  | 1576 | -0.279 | -0.7273 | No |
| 47 | IQGAP3 |  |  | 1584 | -0.282 | -0.7296 | No |
| 48 | SRRM2 |  |  | 1587 | -0.283 | -0.7293 | No |
| 49 | ZNF704 |  |  | 1588 | -0.284 | -0.7279 | No |
| 50 | PRKDC |  |  | 1594 | -0.285 | -0.7291 | No |
| 51 | RCC1 |  |  | 1604 | -0.286 | -0.7324 | No |
| 52 | CCDC150 |  |  | 1605 | -0.286 | -0.7310 | No |
| 53 | CNTRL |  |  | 1610 | -0.288 | -0.7317 | No |
| 54 | HP1BP3 |  |  | 1618 | -0.290 | -0.7340 | No |
| 55 | TMEM97 |  |  | 1629 | -0.293 | -0.7378 | No |
| 56 | SRRM1 |  |  | 1630 | -0.294 | -0.7363 | No |
| 57 | HES6 |  |  | 1632 | -0.294 | -0.7354 | No |
| 58 | GINS1 |  |  | 1634 | -0.295 | -0.7345 | No |
| 59 | RPL8 |  |  | 1647 | -0.299 | -0.7393 | No |
| 60 | ILDR2 |  |  | 1650 | -0.300 | -0.7389 | No |
| 61 | PARP2 |  |  | 1665 | -0.305 | -0.7447 | No |
| 62 | ANAPC11 |  |  | 1667 | -0.306 | -0.7438 | No |
| 63 | IPO5 |  |  | 1672 | -0.308 | -0.7444 | No |
| 64 | ENO1 |  |  | 1673 | -0.308 | -0.7429 | No |
| 65 | RDX |  |  | 1678 | -0.310 | -0.7434 | No |
| 66 | FADS1 |  |  | 1682 | -0.312 | -0.7435 | No |
| 67 | CTPS1 |  |  | 1692 | -0.316 | -0.7466 | No |
| 68 | MGME1 |  |  | 1697 | -0.317 | -0.7472 | Yes |
| 69 | ZNF93 |  |  | 1699 | -0.318 | -0.7462 | Yes |
| 70 | PRIM1 |  |  | 1703 | -0.320 | -0.7462 | Yes |
| 71 | H1FX |  |  | 1705 | -0.321 | -0.7451 | Yes |
| 72 | SNRPC |  |  | 1707 | -0.321 | -0.7441 | Yes |
| 73 | HAUS1 |  |  | 1708 | -0.322 | -0.7425 | Yes |
| 74 | NEDD1 |  |  | 1710 | -0.322 | -0.7415 | Yes |
| 75 | CENPQ |  |  | 1711 | -0.322 | -0.7399 | Yes |
| 76 | PARP1 |  |  | 1713 | -0.323 | -0.7389 | Yes |
| 77 | SASS6 |  |  | 1719 | -0.325 | -0.7399 | Yes |
| 78 | CDC25C |  |  | 1724 | -0.327 | -0.7404 | Yes |
| 79 | PPP2R3C |  |  | 1736 | -0.335 | -0.7445 | Yes |
| 80 | MCM3 |  |  | 1738 | -0.335 | -0.7434 | Yes |
| 81 | PLK4 |  |  | 1740 | -0.336 | -0.7423 | Yes |
| 82 | RMI1 |  |  | 1743 | -0.337 | -0.7417 | Yes |
| 83 | STIL |  |  | 1744 | -0.337 | -0.7401 | Yes |
| 84 | EXOSC9 |  |  | 1746 | -0.338 | -0.7390 | Yes |
| 85 | GLO1 |  |  | 1755 | -0.342 | -0.7415 | Yes |
| 86 | PAICS |  |  | 1757 | -0.342 | -0.7403 | Yes |
| 87 | RAB13 |  |  | 1761 | -0.345 | -0.7402 | Yes |
| 88 | ZNF90 |  |  | 1763 | -0.345 | -0.7391 | Yes |
| 89 | SUZ12 |  |  | 1766 | -0.347 | -0.7384 | Yes |
| 90 | RAN |  |  | 1772 | -0.350 | -0.7393 | Yes |
| 91 | TOPBP1 |  |  | 1781 | -0.354 | -0.7418 | Yes |
| 92 | WDR76 |  |  | 1783 | -0.355 | -0.7406 | Yes |
| 93 | BRIP1 |  |  | 1790 | -0.357 | -0.7420 | Yes |
| 94 | ILF2 |  |  | 1795 | -0.360 | -0.7423 | Yes |
| 95 | LIG1 |  |  | 1797 | -0.361 | -0.7411 | Yes |
| 96 | HNRNPC |  |  | 1804 | -0.362 | -0.7425 | Yes |
| 97 | CENPL |  |  | 1805 | -0.362 | -0.7407 | Yes |
| 98 | TOMM5 |  |  | 1818 | -0.374 | -0.7451 | Yes |
| 99 | NUP50 |  |  | 1823 | -0.376 | -0.7454 | Yes |
| 100 | XRCC6 |  |  | 1825 | -0.377 | -0.7441 | Yes |
| 101 | LDHA |  |  | 1833 | -0.380 | -0.7459 | Yes |
| 102 | CDC45 |  |  | 1836 | -0.382 | -0.7451 | Yes |
| 103 | BARD1 |  |  | 1839 | -0.384 | -0.7443 | Yes |
| 104 | MASTL |  |  | 1843 | -0.385 | -0.7440 | Yes |
| 105 | FANCA |  |  | 1844 | -0.385 | -0.7421 | Yes |
| 106 | MMS22L |  |  | 1846 | -0.387 | -0.7407 | Yes |
| 107 | CCT6A |  |  | 1848 | -0.387 | -0.7394 | Yes |
| 108 | SRSF10 |  |  | 1851 | -0.390 | -0.7385 | Yes |
| 109 | PCNT |  |  | 1854 | -0.392 | -0.7377 | Yes |
| 110 | CEP135 |  |  | 1855 | -0.393 | -0.7357 | Yes |
| 111 | E2F8 |  |  | 1864 | -0.396 | -0.7380 | Yes |
| 112 | EXOSC8 |  |  | 1865 | -0.397 | -0.7361 | Yes |
| 113 | CHD7 |  |  | 1874 | -0.400 | -0.7383 | Yes |
| 114 | SRSF1 |  |  | 1879 | -0.405 | -0.7384 | Yes |
| 115 | GTPBP4 |  |  | 1880 | -0.409 | -0.7364 | Yes |
| 116 | CENPO |  |  | 1884 | -0.410 | -0.7360 | Yes |
| 117 | RBMX |  |  | 1885 | -0.412 | -0.7340 | Yes |
| 118 | SHCBP1 |  |  | 1886 | -0.412 | -0.7320 | Yes |
| 119 | RFC4 |  |  | 1887 | -0.413 | -0.7300 | Yes |
| 120 | SEPT10 |  |  | 1888 | -0.414 | -0.7280 | Yes |
| 121 | POLQ |  |  | 1891 | -0.417 | -0.7270 | Yes |
| 122 | YBX1 |  |  | 1892 | -0.417 | -0.7250 | Yes |
| 123 | SAE1 |  |  | 1893 | -0.417 | -0.7230 | Yes |
| 124 | ASF1B |  |  | 1897 | -0.419 | -0.7225 | Yes |
| 125 | RAD18 |  |  | 1898 | -0.419 | -0.7204 | Yes |
| 126 | E2F3 |  |  | 1907 | -0.429 | -0.7225 | Yes |
| 127 | BAZ1B |  |  | 1910 | -0.429 | -0.7215 | Yes |
| 128 | HNRNPH3 |  |  | 1911 | -0.429 | -0.7194 | Yes |
| 129 | MNS1 |  |  | 1913 | -0.430 | -0.7178 | Yes |
| 130 | HMGA1 |  |  | 1915 | -0.433 | -0.7163 | Yes |
| 131 | POLD3 |  |  | 1918 | -0.436 | -0.7152 | Yes |
| 132 | UHRF1 |  |  | 1921 | -0.438 | -0.7141 | Yes |
| 133 | DIAPH3 |  |  | 1924 | -0.439 | -0.7130 | Yes |
| 134 | HMGXB4 |  |  | 1928 | -0.441 | -0.7124 | Yes |
| 135 | GINS2 |  |  | 1932 | -0.445 | -0.7118 | Yes |
| 136 | LINC01224 |  |  | 1934 | -0.446 | -0.7102 | Yes |
| 137 | SNRPB |  |  | 1937 | -0.447 | -0.7091 | Yes |
| 138 | HNRNPDL |  |  | 1940 | -0.448 | -0.7079 | Yes |
| 139 | MZT2B |  |  | 1941 | -0.449 | -0.7058 | Yes |
| 140 | TTF2 |  |  | 1942 | -0.449 | -0.7036 | Yes |
| 141 | STAG1 |  |  | 1943 | -0.450 | -0.7014 | Yes |
| 142 | CCNF |  |  | 1947 | -0.451 | -0.7008 | Yes |
| 143 | CLIC1 |  |  | 1949 | -0.454 | -0.6991 | Yes |
| 144 | NAP1L1 |  |  | 1951 | -0.455 | -0.6974 | Yes |
| 145 | DDX11 |  |  | 1952 | -0.456 | -0.6952 | Yes |
| 146 | NOP56 |  |  | 1953 | -0.456 | -0.6930 | Yes |
| 147 | RFC2 |  |  | 1954 | -0.457 | -0.6907 | Yes |
| 148 | DDX39A |  |  | 1957 | -0.458 | -0.6896 | Yes |
| 149 | DDIAS |  |  | 1960 | -0.460 | -0.6884 | Yes |
| 150 | ZWILCH |  |  | 1962 | -0.461 | -0.6867 | Yes |
| 151 | RNASEH2C |  |  | 1964 | -0.462 | -0.6849 | Yes |
| 152 | SEPHS1 |  |  | 1967 | -0.464 | -0.6837 | Yes |
| 153 | DNMT1 |  |  | 1969 | -0.465 | -0.6820 | Yes |
| 154 | TIMM10 |  |  | 1975 | -0.470 | -0.6823 | Yes |
| 155 | CMC2 |  |  | 1977 | -0.473 | -0.6805 | Yes |
| 156 | CIT |  |  | 1978 | -0.474 | -0.6782 | Yes |
| 157 | C19orf48 |  |  | 1979 | -0.474 | -0.6759 | Yes |
| 158 | HAUS6 |  |  | 1984 | -0.478 | -0.6757 | Yes |
| 159 | CSE1L |  |  | 1985 | -0.479 | -0.6734 | Yes |
| 160 | SUPT16H |  |  | 1986 | -0.479 | -0.6711 | Yes |
| 161 | SACS |  |  | 1987 | -0.480 | -0.6687 | Yes |
| 162 | NASP |  |  | 1988 | -0.481 | -0.6664 | Yes |
| 163 | TIMELESS |  |  | 1989 | -0.482 | -0.6640 | Yes |
| 164 | CDC6 |  |  | 1991 | -0.483 | -0.6622 | Yes |
| 165 | MCM4 |  |  | 1993 | -0.487 | -0.6604 | Yes |
| 166 | SKA1 |  |  | 1996 | -0.490 | -0.6590 | Yes |
| 167 | SPAG5 |  |  | 1997 | -0.490 | -0.6567 | Yes |
| 168 | TRA2B |  |  | 1998 | -0.490 | -0.6543 | Yes |
| 169 | PSMC3 |  |  | 1999 | -0.492 | -0.6519 | Yes |
| 170 | FAM111A |  |  | 2002 | -0.497 | -0.6505 | Yes |
| 171 | TK1 |  |  | 2003 | -0.497 | -0.6481 | Yes |
| 172 | LRR1 |  |  | 2004 | -0.497 | -0.6457 | Yes |
| 173 | TUBB6 |  |  | 2006 | -0.500 | -0.6438 | Yes |
| 174 | HNRNPUL1 |  |  | 2009 | -0.501 | -0.6424 | Yes |
| 175 | CDK5RAP2 |  |  | 2012 | -0.503 | -0.6410 | Yes |
| 176 | CHEK1 |  |  | 2013 | -0.504 | -0.6385 | Yes |
| 177 | SIVA1 |  |  | 2014 | -0.505 | -0.6361 | Yes |
| 178 | HIRIP3 |  |  | 2017 | -0.508 | -0.6347 | Yes |
| 179 | FEN1 |  |  | 2018 | -0.511 | -0.6322 | Yes |
| 180 | SMC1A |  |  | 2021 | -0.513 | -0.6307 | Yes |
| 181 | RRM1 |  |  | 2023 | -0.516 | -0.6288 | Yes |
| 182 | RANBP1 |  |  | 2024 | -0.517 | -0.6262 | Yes |
| 183 | HAUS8 |  |  | 2026 | -0.517 | -0.6243 | Yes |
| 184 | PARPBP |  |  | 2030 | -0.520 | -0.6233 | Yes |
| 185 | DEPDC1B |  |  | 2037 | -0.527 | -0.6239 | Yes |
| 186 | HELLS |  |  | 2038 | -0.528 | -0.6213 | Yes |
| 187 | TPM4 |  |  | 2039 | -0.528 | -0.6187 | Yes |
| 188 | CDCA3 |  |  | 2041 | -0.529 | -0.6167 | Yes |
| 189 | NCL |  |  | 2045 | -0.532 | -0.6157 | Yes |
| 190 | TTK |  |  | 2047 | -0.533 | -0.6136 | Yes |
| 191 | NCAPD3 |  |  | 2051 | -0.537 | -0.6126 | Yes |
| 192 | ILF3 |  |  | 2052 | -0.537 | -0.6100 | Yes |
| 193 | KIF22 |  |  | 2053 | -0.541 | -0.6073 | Yes |
| 194 | CENPJ |  |  | 2054 | -0.544 | -0.6047 | Yes |
| 195 | CKLF |  |  | 2055 | -0.544 | -0.6020 | Yes |
| 196 | SRSF7 |  |  | 2056 | -0.544 | -0.5994 | Yes |
| 197 | CTNNAL1 |  |  | 2059 | -0.548 | -0.5978 | Yes |
| 198 | SNRPE |  |  | 2062 | -0.565 | -0.5961 | Yes |
| 199 | STMN1 |  |  | 2063 | -0.567 | -0.5933 | Yes |
| 200 | PTMA |  |  | 2065 | -0.570 | -0.5911 | Yes |
| 201 | DTL |  |  | 2066 | -0.573 | -0.5883 | Yes |
| 202 | FANCD2 |  |  | 2068 | -0.577 | -0.5860 | Yes |
| 203 | ZWINT |  |  | 2069 | -0.577 | -0.5832 | Yes |
| 204 | CEP152 |  |  | 2070 | -0.578 | -0.5804 | Yes |
| 205 | RTKN2 |  |  | 2071 | -0.579 | -0.5776 | Yes |
| 206 | RACGAP1 |  |  | 2072 | -0.579 | -0.5748 | Yes |
| 207 | MND1 |  |  | 2074 | -0.586 | -0.5725 | Yes |
| 208 | SKA3 |  |  | 2077 | -0.590 | -0.5706 | Yes |
| 209 | BUB3 |  |  | 2078 | -0.596 | -0.5677 | Yes |
| 210 | AURKB |  |  | 2079 | -0.598 | -0.5648 | Yes |
| 211 | GGH |  |  | 2080 | -0.603 | -0.5619 | Yes |
| 212 | SRSF3 |  |  | 2081 | -0.606 | -0.5590 | Yes |
| 213 | CENPK |  |  | 2082 | -0.607 | -0.5560 | Yes |
| 214 | HNRNPR |  |  | 2083 | -0.612 | -0.5531 | Yes |
| 215 | USP1 |  |  | 2084 | -0.612 | -0.5501 | Yes |
| 216 | CKAP5 |  |  | 2086 | -0.623 | -0.5476 | Yes |
| 217 | NCAPD2 |  |  | 2089 | -0.627 | -0.5456 | Yes |
| 218 | HNRNPU |  |  | 2090 | -0.628 | -0.5425 | Yes |
| 219 | SSRP1 |  |  | 2091 | -0.630 | -0.5395 | Yes |
| 220 | MCM8 |  |  | 2092 | -0.635 | -0.5364 | Yes |
| 221 | KIF18A |  |  | 2093 | -0.636 | -0.5333 | Yes |
| 222 | FAM111B |  |  | 2094 | -0.637 | -0.5302 | Yes |
| 223 | SET |  |  | 2095 | -0.639 | -0.5271 | Yes |
| 224 | DNAJC9 |  |  | 2097 | -0.641 | -0.5245 | Yes |
| 225 | RFC3 |  |  | 2098 | -0.642 | -0.5214 | Yes |
| 226 | BRCA2 |  |  | 2100 | -0.650 | -0.5188 | Yes |
| 227 | ITGB3BP |  |  | 2102 | -0.652 | -0.5161 | Yes |
| 228 | NCAPH |  |  | 2104 | -0.652 | -0.5135 | Yes |
| 229 | CKAP2L |  |  | 2109 | -0.663 | -0.5123 | Yes |
| 230 | ALYREF |  |  | 2111 | -0.677 | -0.5096 | Yes |
| 231 | RPA3 |  |  | 2112 | -0.678 | -0.5063 | Yes |
| 232 | LSM3 |  |  | 2114 | -0.694 | -0.5034 | Yes |
| 233 | CKS2 |  |  | 2115 | -0.694 | -0.5001 | Yes |
| 234 | RAD21 |  |  | 2118 | -0.697 | -0.4977 | Yes |
| 235 | ANP32B |  |  | 2119 | -0.700 | -0.4943 | Yes |
| 236 | CDCA2 |  |  | 2120 | -0.705 | -0.4909 | Yes |
| 237 | SRSF2 |  |  | 2122 | -0.706 | -0.4880 | Yes |
| 238 | PA2G4 |  |  | 2123 | -0.707 | -0.4846 | Yes |
| 239 | CENPM |  |  | 2125 | -0.709 | -0.4816 | Yes |
| 240 | HNRNPD |  |  | 2126 | -0.713 | -0.4782 | Yes |
| 241 | RAD51AP1 |  |  | 2127 | -0.715 | -0.4747 | Yes |
| 242 | CDCA8 |  |  | 2129 | -0.722 | -0.4717 | Yes |
| 243 | CKAP2 |  |  | 2131 | -0.729 | -0.4687 | Yes |
| 244 | ATAD5 |  |  | 2133 | -0.742 | -0.4656 | Yes |
| 245 | VRK1 |  |  | 2134 | -0.743 | -0.4620 | Yes |
| 246 | KIF2C |  |  | 2136 | -0.756 | -0.4589 | Yes |
| 247 | BRCA1 |  |  | 2138 | -0.758 | -0.4557 | Yes |
| 248 | KIF14 |  |  | 2141 | -0.773 | -0.4530 | Yes |
| 249 | NDC80 |  |  | 2142 | -0.775 | -0.4492 | Yes |
| 250 | H2AFV |  |  | 2143 | -0.779 | -0.4454 | Yes |
| 251 | FBXO5 |  |  | 2144 | -0.779 | -0.4417 | Yes |
| 252 | KIF23 |  |  | 2145 | -0.782 | -0.4379 | Yes |
| 253 | GMNN |  |  | 2146 | -0.785 | -0.4340 | Yes |
| 254 | LMNB2 |  |  | 2147 | -0.786 | -0.4302 | Yes |
| 255 | MELK |  |  | 2148 | -0.786 | -0.4264 | Yes |
| 256 | ANLN |  |  | 2149 | -0.797 | -0.4225 | Yes |
| 257 | SMC3 |  |  | 2150 | -0.800 | -0.4187 | Yes |
| 258 | DEK |  |  | 2154 | -0.817 | -0.4163 | Yes |
| 259 | DUT |  |  | 2155 | -0.824 | -0.4123 | Yes |
| 260 | NCAPG2 |  |  | 2156 | -0.827 | -0.4082 | Yes |
| 261 | TUBB |  |  | 2157 | -0.831 | -0.4042 | Yes |
| 262 | NUCKS1 |  |  | 2158 | -0.832 | -0.4002 | Yes |
| 263 | LSM5 |  |  | 2159 | -0.835 | -0.3961 | Yes |
| 264 | MCM7 |  |  | 2160 | -0.839 | -0.3920 | Yes |
| 265 | TUBB4B |  |  | 2162 | -0.843 | -0.3885 | Yes |
| 266 | HMGB3 |  |  | 2164 | -0.852 | -0.3849 | Yes |
| 267 | KIF20B |  |  | 2165 | -0.852 | -0.3807 | Yes |
| 268 | SPC24 |  |  | 2166 | -0.853 | -0.3766 | Yes |
| 269 | ARHGAP11A |  |  | 2167 | -0.855 | -0.3724 | Yes |
| 270 | MCM10 |  |  | 2169 | -0.858 | -0.3688 | Yes |
| 271 | FOXM1 |  |  | 2171 | -0.861 | -0.3651 | Yes |
| 272 | CDCA5 |  |  | 2172 | -0.866 | -0.3609 | Yes |
| 273 | LBR |  |  | 2173 | -0.870 | -0.3567 | Yes |
| 274 | BUB1B |  |  | 2174 | -0.871 | -0.3525 | Yes |
| 275 | NUF2 |  |  | 2176 | -0.877 | -0.3487 | Yes |
| 276 | CCDC34 |  |  | 2180 | -0.898 | -0.3459 | Yes |
| 277 | CHAF1A |  |  | 2182 | -0.903 | -0.3421 | Yes |
| 278 | MIS18BP1 |  |  | 2183 | -0.903 | -0.3377 | Yes |
| 279 | KIF15 |  |  | 2185 | -0.915 | -0.3338 | Yes |
| 280 | KIF4A |  |  | 2186 | -0.921 | -0.3293 | Yes |
| 281 | PRR11 |  |  | 2187 | -0.930 | -0.3248 | Yes |
| 282 | ORC6 |  |  | 2188 | -0.937 | -0.3202 | Yes |
| 283 | C21orf58 |  |  | 2189 | -0.937 | -0.3157 | Yes |
| 284 | CENPW |  |  | 2192 | -0.943 | -0.3121 | Yes |
| 285 | TUBA1B |  |  | 2193 | -0.947 | -0.3075 | Yes |
| 286 | LMNB1 |  |  | 2194 | -0.950 | -0.3029 | Yes |
| 287 | HJURP |  |  | 2195 | -0.962 | -0.2983 | Yes |
| 288 | UBE2T |  |  | 2196 | -0.965 | -0.2936 | Yes |
| 289 | FANCI |  |  | 2198 | -0.966 | -0.2894 | Yes |
| 290 | CCNB2 |  |  | 2200 | -0.983 | -0.2851 | Yes |
| 291 | CKS1B |  |  | 2201 | -0.987 | -0.2804 | Yes |
| 292 | HIST1H1E |  |  | 2202 | -0.992 | -0.2755 | Yes |
| 293 | ESCO2 |  |  | 2204 | -1.004 | -0.2712 | Yes |
| 294 | SMC2 |  |  | 2205 | -1.006 | -0.2663 | Yes |
| 295 | KIF11 |  |  | 2206 | -1.008 | -0.2614 | Yes |
| 296 | EZH2 |  |  | 2207 | -1.017 | -0.2565 | Yes |
| 297 | SPC25 |  |  | 2208 | -1.019 | -0.2515 | Yes |
| 298 | XRCC2 |  |  | 2210 | -1.028 | -0.2470 | Yes |
| 299 | CENPE |  |  | 2211 | -1.036 | -0.2420 | Yes |
| 300 | DHFR |  |  | 2212 | -1.040 | -0.2370 | Yes |
| 301 | NUDT1 |  |  | 2214 | -1.054 | -0.2324 | Yes |
| 302 | MYBL2 |  |  | 2215 | -1.058 | -0.2272 | Yes |
| 303 | CLSPN |  |  | 2216 | -1.060 | -0.2221 | Yes |
| 304 | CENPN |  |  | 2217 | -1.079 | -0.2168 | Yes |
| 305 | UBE2C |  |  | 2218 | -1.081 | -0.2116 | Yes |
| 306 | CENPU |  |  | 2219 | -1.088 | -0.2063 | Yes |
| 307 | H2AFZ |  |  | 2220 | -1.090 | -0.2010 | Yes |
| 308 | HMGB1 |  |  | 2221 | -1.116 | -0.1956 | Yes |
| 309 | NCAPG |  |  | 2222 | -1.122 | -0.1901 | Yes |
| 310 | H2AFX |  |  | 2223 | -1.146 | -0.1846 | Yes |
| 311 | CCNB1 |  |  | 2224 | -1.148 | -0.1790 | Yes |
| 312 | CDK1 |  |  | 2225 | -1.153 | -0.1734 | Yes |
| 313 | PTTG1 |  |  | 2226 | -1.198 | -0.1676 | Yes |
| 314 | ANP32E |  |  | 2227 | -1.200 | -0.1618 | Yes |
| 315 | MAD2L1 |  |  | 2228 | -1.204 | -0.1559 | Yes |
| 316 | GTSE1 |  |  | 2230 | -1.220 | -0.1505 | Yes |
| 317 | DBF4 |  |  | 2232 | -1.247 | -0.1450 | Yes |
| 318 | HMGN2 |  |  | 2233 | -1.267 | -0.1388 | Yes |
| 319 | RRM2 |  |  | 2234 | -1.268 | -0.1327 | Yes |
| 320 | SMC4 |  |  | 2236 | -1.281 | -0.1270 | Yes |
| 321 | PBK |  |  | 2237 | -1.325 | -0.1205 | Yes |
| 322 | ATAD2 |  |  | 2238 | -1.381 | -0.1138 | Yes |
| 323 | TMPO |  |  | 2239 | -1.382 | -0.1071 | Yes |
| 324 | PRC1 |  |  | 2240 | -1.397 | -0.1003 | Yes |
| 325 | HMGB2 |  |  | 2241 | -1.469 | -0.0932 | Yes |
| 326 | BIRC5 |  |  | 2242 | -1.469 | -0.0861 | Yes |
| 327 | HIST1H1C |  |  | 2243 | -1.485 | -0.0788 | Yes |
| 328 | ASPM |  |  | 2244 | -1.490 | -0.0716 | Yes |
| 329 | TOP2A |  |  | 2246 | -1.562 | -0.0645 | Yes |
| 330 | NUSAP1 |  |  | 2247 | -1.600 | -0.0568 | Yes |
| 331 | TPX2 |  |  | 2248 | -1.609 | -0.0490 | Yes |
| 332 | CENPF |  |  | 2249 | -1.691 | -0.0408 | Yes |
| 333 | HIST1H1D |  |  | 2250 | -1.720 | -0.0324 | Yes |
| 334 | HIST1H1B |  |  | 2251 | -1.796 | -0.0237 | Yes |
| 335 | MKI67 |  |  | 2252 | -1.892 | -0.0145 | Yes |
| 336 | HIST1H4C |  |  | 2253 | -2.984 | 0.0000 | Yes |
Table: GSEA details [plain text format]

  

Fig 2: RICHARDS\_NOWAKOWSKI\_SCIENCE\_2017\_MGE\_DIV\_UPREG: Random ES distribution      
 Gene set null distribution of ES for **RICHARDS\_NOWAKOWSKI\_SCIENCE\_2017\_MGE\_DIV\_UPREG**
